# Abstracts and Gleanings

**Published:** 1883-07-20

**Authors:** 


### ABSTRACTS AND GLEANINGS.

**Bromide of Sodium and Bromide of Potassium.**—A physiological study of the two salts in question supports the following propositions:

1. The bromide of sodium, because it is a sodic compound, should be more congenial, less disturbing, to the fluids and solids of the body than its potassic congener.

2. The sodium salt, in extended use, should be less depressing to the heart, all potassic salts, after a time, tending to produce cardiac depression.

3. The sodic bromide is less offensive to the taste, much less irritating to the stomach.

4. The bromide of sodium should have equal, if not superior, general therapeutic power with the bromide of potassium, since while the former has a bromine per cent. of 78, the latter has but 66.

To which it may be added, my clinical experience has brought me the following conclusions:

1. The bromide of sodium has equal therapeutic power, throughout the entire range of medication (with possibly an exception), with that of the bromide of potassium.

2. Not only this, but the bromide of sodium has superior therapeutic value, both from the greater mildness of its physiological impression, and because of additional therapeutic applications which were confined to the potassic salt, would be inconvenient if not impossible.

And, first, bromide of sodium is pre-eminently the child's bromide, as tannate of quinia is the "babe's quinine." It is much less disagreeable to the taste, and less likely to be objected to. In the case of the very young, children a few months or under two years of age, where small quantities of the remedy are all that is required, I have frequently followed the suggestion of the French, and seasoned the infant's food with the bromide of sodium instead of with common salt, for example, a few grains added to the bottle of milk several times through the day or at bed-time. The occasions when we must disgust the young with offensive and bulky remedies are far too many; it is a good thing to avoid them when we can. Again, the babe will seldom object to two or four grains of the sodic bromide in a teaspoonful of water, sweetened or not. The bromide of potassium is another matter altogether.

For nausea and vomiting of the adult, and especially in the nervous female, occasioned by whatever common derangement of stomach or reflected disturbance, I have found bromide of sodium in ice-water—a half drachm to the half tumbler—one of the most effective of remedies. It must, of course, be drank slowly, a little at a time, as the stomach can receive it, and it is an essential condition that a little ice be kept in the solution until it is all taken. I remember in one instance treating effectively three such cases in the course of the week. They were all night calls, but I avoided going out by sending the bromide with instructions how to use it, and, in one case, also ice from my refrigerator. I know of no other one remedy, not even morphia, in minute doses, that will accomplish so much, and morphia may be objectionable on account of after-effect, which the bromide never is. I should despair of treating this class of cases with bromide of potassium ; the taste would often insure its refusal almost before it had reached the stomach—in a case where the vomitive tract of impression and conduction involves not alone the surface of the stomach, but also œsophagus, fauces, and even to the very mouth—or, if the stomach could be induced to receive it, its positively irritating effect would soon occasion its rejection, and leave the patient worse than before any medicine was taken. Similar criticism applies to the choice of a bromide for sea-sickness.—*Boston Med. and Surg. Jour.*

**Boroglyceride in the Treatment of Diseases of the Eye and Ear.**—L. Webster Fox, M. D., in the Western Medical Reporter, says :

Boroglyceride, which has drawn the attention of surgeons to its antiseptic and astringent properties, has been used by me in the ophthalmological and aural department of the Germantown Hospital for the last six months. The number of cases treated have been numerous enough to justify my recommending it as a very valuable agent in the treatment of certain ocular and aural troubles.

To Dr. Granville Faught, formerly resident physician, I am greatly indebted for the successful preparation of the drug, as well as to his careful attention to the patients treated, and notes made on each respective case. I refer the reader to Dr. Faught's article on boroglyceride, in the present number of this journal, not only as to the manner of its preparation, but also to his views on the drug as a therapeutic remedy.

Boroglyceride, when applied to the healthy conjunctiva, produces a sharp, smarting pain, lasting several minutes, profuse lachrymation and congestion of the smaller veins and arteries, not only of the mucous surface of the lids, but of the conjunctiva covering the eyeball ; this being followed by contraction of the vessels, the sclerotic becoming pearly, and cornea particularly brilliant.

It has an acrid taste, not unpleasant. Its astringent properties are made manifest by a decided puckering of the mucous lining of the mouth.

In certain chronic conditions of the conjunctiva, patients do not complain of the smarting pain at first, but after several days' use this becomes manifest.

The preparations used have contained ten per cent., and fifty per cent. of the drug in glycerine. It is soluble in glycerine, cold and hot water.

In cases of acute conjunctivitis, with slight secretions, the following collyrium has been found efficacious:

℞ Boroglyceride.................................. ʒ j,

Camphor or rose water...................... } aa ℥ j.

Distilled water.............................. }

This can be applied either by a bit of cotton-wool or by the spray. After several applications, the congestion disappears, and the mucous surface is restored to its normal condition. In granular lids, with much thickening of the conjunctiva complicated with pannus of cornea, a fifty per cent. solution was used. The slight purulent secretions were checked immediately; the thickened and congested condition of the conjunctiva was reduced, the vessels on the cornea disappeared leaving it clear and transparent; but no change is observable in the hypertrophied condition of the papillæ, excepting to make them more pronounced in their outline. At this stage of the treatment, xerosis conjunctivæ is not unfrequently produced. The conjunctivæ are free from moisture, and the patient has sensations of heat and dryness in the eyes, which are distressing. The treatment should be discontinued, and a solution of nitrate of silver, five or ten grains to the ounce, substituted; one application daily should be made to the parts, until the normal secretions are restored; then the sulphate of copper, in substance, is to be applied once daily to the now reduced trachoma. Under this treatment the granulations disappear very rapidly, the lids become smooth, and, where there is pannus, the cornea regains and retains its transparency.— *Western Med. Reporter.*

**Hemorrhagic Diathesis.**—Dr. Wm. Savery, in Obstetric Society of Philadelphia, related the history of a boy, five years of age, who had fallen and received a slight wound of the scalp from a nail sticking out of a post; it was a mere scratch and did not need a stitch to hold it together, but it bled profusely; all sorts of domestic remedies, including cob-web, had been tried without avail. The doctor finally succeeded with lint, wet with Monsel's solution and continued firm pressure.

A few days later the same boy fell from one step on the floor; there was no external wound nor loss of blood, but the side of the face was enormously swelled from hemorrhage into the tissues. A course of iron and tonics has improved the boy's appearance, but he is still pale.

Dr. Horace Williams had seen a case of obstinate and prolonged hemorrhage after the extraction of a tooth; it was finally stopped by fitting a cork into the alveolar cavity.

An infant aged nine days was attacked with purpuric spots over the body and bleeding at the navel. To the latter were applied successively styptic colloid, tannin, Monsel's solution, Monsel's salt in powder, and finally transverse pains and figure of eight ligatures: but the bleeding reappeared as soon as the latter came away and the child finally died from loss of blood.

Dr. R. A. Cleemann had under his care a young man who had previously suffered from profuse hemorrhage for two days, consequent on the extraction of a tooth; the hemorrhage was finally stopped by Dr. Hartshorn, who plugged the cavity with a styptic

Dr. H. advised the young man never again to run the risk of a hemorrhage of any kind, as it would probably prove fatal. Recently, he had been suffering severely from a toothache which nothing but extraction could relieve. Dr. Cleemann put him on gallic acid internally, and tannic acid locally, for two weeks before the extraction, which was accomplished without any unusual loss of blood.

In a case of nasal hemorrhage, the anterior and posterior nares were plugged, but then ecchymoses appeared around the eyes, and the plugs were removed, transfusion of a few ounces of blood was employed and the hemorrhage ceased and did not return ; the patient died three months later of phthisis.

Dr. E. L. Duer considered gallic acid a very valuable remedy for hemorrhage. He would like particularly to bring before the Society the old but neglected remedy, Erigeron or flea-bane ; the tincture and the volatile oil are very efficient when used internally to stop hemorrhage. The oil may be given in doses of ten drops every ten minutes until the bleeding is checked, after which it may be continued at longer intervals until the tendency has passed away.

Dr. Githens had been using oil of Erigeron for a number of years with remarkable success as an internal hemostatic. It was far more reliable than any other with which he was acquainted.—*Obstetrie Gazette.*

**Viability of the Premature Fetus.**—At the meeting of the Lebanon, Ohio, Medical Society some months ago, Dr. Scoville related a case with essentially the following outline of history :

The patient in question menstruated during the second week of last December ; claims to have positive knowledge that she conceived December 23d. She was confined with a premature child on the 29th of May, which would make a period of 155 days. The child lived 23 hours ; cried several times ; took nourishment. It was a male, weight, one pound twelve ounces. Length, about twelve inches.

Such cases of viability of the premature fetus come to us from time to time, and they suggest the inquiry as to how early we are to deem premature children capable, not merely of temporary viability, but of going forward to live.

Not long since, I found in one of the medical journals a case much the same in history with that of Dr. Scoville ; but the time is still shorter. It is reported by Dr. Williams, of Frankfort, Kentucky.

"December 23d, 1882, Mrs. S. aborted ; was unwell twice afterward ; well February 13th, 1882, of the last menstruation. July 4th, the doctor attended her again in an abortion, *i.e.*, 141 days from the last menstruation. The fetus weighed one and one-half pounds ; moved and breathed for half an hour and made a feeble cry.

Such cases as these do not prove that with special favoring circumstances or care, the child might or might not have lived ; and yet there is suggested to us, that with so much matured organism and vitality a child might very frequently survive ; and if so, what means should be employed when these favorable cases of prematurity occur.

Physiologically, however, the sentiment of the profession has not favored the probability of sustaining the life of children born at a stage of prematurity at all expressed in such cases as the two I have briefly quoted.

The general formula announced in most of our old and standard authorities was to the effect that about seven lunar months, or something less than 200 days, of the intra-uterine existence and development, was necessary to reach a stage of independent and permanent viability.—*Obstetric Gazette.*

**Percussion of the Colon in Diarrhœa.**—Diarrhœa depending upon fecal accumulations in the lower bowel (diarrhœa paradoxa) is a well known condition, the treatment of which by laxatives is of long-recognized utility. The diagnosis, however, between this form of diarrhœa and that other whose location is, more strictly speaking, in the small intestine, has often presented considerable difficulty. In an article upon this subject, in the Deutsche Medicinische Wochenschrift of February 14, 1883, Dr. Goedicke advocates the systematic practice of abdominal percussion in all cases of diarrhœa. He was led to adopt the practice in this wise: Several years before, when a young army surgeon, his suspicions were often aroused by the number of soldiers coming to him with the complaint of diarrhœa. In order to detect the malingerers, he made use of percussion of the colon, reasoning that in genuine diarrhœa the descending colon should be empty, and therefore give a tympanitic percussion note. He was surprised, therefore, to find that the contrary was usually the case. In most of the men in whom diarrhœa actually existed, as was ascertained from the reports of the infirmary orderlies, the percussion note of the descending colon was dull. The investigations now undertaken led him to the following conclusions: 1. In a healthy individual, with normal movements, if we percuss the colon we shall find that the left iliac fossa usually gives a flatter note than the right. 2. In patients suffering from diarrhœa the greater dullness may be on either side, but is usually, in otherwise healthy persons, on the left. 3. The same condition obtains in children. 4. Whenever there is tenderness on pressure, it is found on the same side as het greater dullness. 5. The term "dullness" is to be understood as relative and not necessarily absolute, for the percussion note on both sides may be actually tympanitic if the colon be distended with gas. The author asserts that by far the more common form of diarrhæa is that excited by fecal accumulation in the large intestine. It is this variety which is characterized by increased relative dullness in the left iliac fossa, and in which opiates and astringent remedies are contra-indicated. In the other form of diarrhœa the trouble is in the upper bowel, and here the percussion note upon the right side is more dull, or less tympanitic, than that on the left. It is in these cases that the ordinary diarrhœa medicines find their application. Dr. Goedicke concludes by urging the practice of abdominal percussion in every case of diarrhæa, where possible (it is always possible in children, and it is in children that the knowledge of the true nature of the trouble is of the greatest importance), and he states his conviction that the more general this practice becomes, the less frequently will opium be employed in the treatment of diarrhœ.—*N. Y. Med. Rec.*

**Iodoform in Spray Form.**—The use of iodoform as applied to wounds as a dressing, by means of the alcohol or ether spray, has been closely investigated by P. G. Unna, of Hamburg. The author's results were so satisfactory with this method of applying drugs, that he used quite a number of remedies in different diseases in the same manner.

The objection to the use of iodoform as a powder, is mainly due to the unavoidable toxic symptoms that arise in nearly all cases where an extensive surface is dusted over. Nor can its action be limited when untoward symptoms have set in, especially if the powder has been thrown into a cavity.

The advantages claimed for the method of dissolving a drug in ether or alcohol, and then applying it by means of a spray, are many and important: 1. The spray has a tendency to relieve pain. 2. The surgeon can reach parts that are inaccessible when a liquid or powder is used. 3. The medicament is not wasted as by the other plans of treatment.

In the following pathological conditions the spray is especially indicated. Inflammation of mucous surfaces (post nares), pharynx, urethra, rectum, and vagina, especially in mucous surfaces where the excretions have a tendency to wash the medicament away. Chrysophanic ether spray may be employed in herpes tonsurans, favus, sycosis parasitica and psoriasis capitis. Hydrate of chloral, in spray form, acts very kindly as a local anæsthetic, where minor operations are to be performed. The collodion is applicable in cases where the drug will not adhere to the surface that must be treated; the remedy is applied first, then coated with the collodion spray, which, by evaporating, forms a covering. The following remedies are soluble in ether or alcohol: Salicylic acid, benzoic acid, acetic acid, chrysophanic acid, pyrogallic acid, citric acid, carbolic acid, atropia, codein, digitalin, santonin and all the alkaloid of cinchona, etc. Boracic acid, gallic acid, arsenic acid, oxalic acid, tannic acid, nitrate of silver, acetate of lead, chloride of zinc, caustic, potash and a few others are soluble in alcohol only. This table is given by the author as a guide, since he thinks the treatment of wounds by the spray has a brilliant future.—*Deutsch Med. Zeit.*

**The Teeth in Diabetes.**—Dr. Magitot has, after an examination of many diabetics, come to the following conclusions: First, examination of the mouths of diabetics furnishes a constant symptom of the disease. Second, this symptom is a lesion of the alveolar border, which may be designated as an alveolar osteo-periostitis. Third, this manifestation appears at the outset of the disease, persists during its course, and can in consequence, be considered a pathognomonic symptom. Fourth, this alveolar affection, considered as a symptom of diabetes, presents three periods. Its first period is that of simple deviation of the teeth. Its second period is that of loosening of the teeth and alveolar catarrh. Each of these periods is in relation to the phase of the constitutional disease. The third period, that of the falling out of the teeth, corresponds to a more advanced state of glycosuria. Besides this last symptom there may occur, if the patient lives long enough, an osseous resorption, which may, or may not, be consecutive to a gangrene of the gums. The appearance of this latter complication is evidence of a critical stage of the disease, as it ordinarily ushers in its fatal termination. The value, as a symptom, of the first stage of dental changes remains to be determined. It must be obvious, however, that it can only occur in the more chronic forms of glycosuric diabetes.—*Independent Practitioner.*

**Diphtheria.**—The following summary of observations is given by Dr. Mulheron in Therapeutic Gazette :

1. Diphtheria may be either local or constitutional in its origin.

2. It may continue as a purely local or as a purely constitutional disease, or the local disease may be followed by constitutional infection, or *vice versa*—the disease in the vast majority of instances manifesting itself in both the constitutional disturbance and the local affection.

3. The comparative value of the local and constitutional remedies is dependent upon the nature of the affection in individual cases.

4. Diphtheria is a contagious disease, but not liable to attack a healthy mucous membrane or to find an entrance through it into the circulation.

5. The contagium of diphtheria is not a micrococcus, nor is it visible under the most powerful microscope yet manufactured.

6. The contagium of diphtheria is of a gaseous nature (the result of decomposing fæcal and other organic matter), and can be neutralized only by a true disinfectant and not by an antiseptic.

7. The best local application is the tincture of the chloride of iron. It may be supplemented by other applications according to the indications in individual cases.

8. In a typical case of asthenic diphtheria, administer large (10 grains) and frequently repeated (hourly) doses of calomel until the characteristic stools are secured. Following this give large doses of the tincture of the chloride of iron every two hours, and administer alcohol within the limits of intoxication. In asthenic cases the calomel should be omitted and the main reliance placed on the iron and alcohol.

**Local Anæsthesia.**—By the following method abscesses, felons, boils, etc., can be opened with little or no pain:

Sharpen to a point a stick about six inches in length. Dip the point into liquified carbolic acid, and apply to the point chosen for opening. After a moment's delay cut the skin with the knife; then take a little of the acid on the point of the stick and apply in the incision with a gentle rotary motion. By frequent applications of the acid, and a gentle rotary motion of the stick persistently applied, an opening can be made to the required depth. The carbolic acid produces first anæsthesia, then death of the parts to which it is applied in the foregoing manner.

I have little doubt but, by patience and perseverance, a stick might be made to pass, without pain, through the entire thickness of the fleshy part of the thigh. The following are cases to the point:

Mr. J. G., a young man, aged 22 years, had passed several sleepless nights, and had suffered great pain, with a deep palmar abscess. He presented himself to me with a worn and haggard expression of countenance, and with his nervous system unstrung. In a few minutes I succeeded in making a free opening, causing little or no pain, through which pus escaped. A rough measurement showed an aperture through the swollen tissues seven-eighths of an inch in depth.

Miss S. S. presented herself to me, under nearly the same circumstances and conditions as in the preceding case, except with felon on her left thumb. A few moments' manipulation relieved her without pain, and left a free opening, through which pus freely escaped. The depth of the opening was a scant three-fourths of an inch.—*Southern Practitioner.*

**Six Years Experience in the Treatment of Syphilis.**—Dr. Charles R. Drysdale thus concludes an article on this subject in the Medical Press: "The treatment of syphilis, commencing with the initial lesion, ought to be continuous, and should consist of very small doses of some mercurial salt, continued for months. When severe symptoms are seen, inunction, calomel vapor baths, or fractional doses of the mercurial salt should be given for a week or so every four hours, in combination with large doses of the iodide of potassium, sodium or ammonium. Atropia drops should be frequently used in iritis. In gummy deposits the chief curative remedy is iodide of potassium in large doses; but a tonic dose of some form of mercurial salt may be added as a germicide. If cerebral symptoms supervene, they are to be treated energetically with the iodide and with immersion.

" Mercury in such small doses seems to do no harm to the genera health, and there is much evidence to show that it is a tonic, which may be given even for years with advantage in some cases of anæmia. All cases of syphilis, mild or severe, should be treated by these small doses of mercury in order to prevent the supervention of tertiary symptoms or gummy products. The germ of syphilis has not yet been seen by the microscope, but it exists in all probability, and this is the rational account of the useful action of mercury and iodine, which are both germicides."—*Medical and Surgical Reporter.*

**Vaccination with Saliva of a Calf.**—Dr. T. J. Reid, of Hot Springs, Ark , writes to the Louisville Medical News: "Quite recently my attention has been called to an accidental vaccination of a respectable lady in this vicinity with the fresh saliva of a calf while sucking. This lady had on the index finger of her right hand two nævi or small warts. While milking her cow the calf annoyed her, and she (lawfullv in this State) gave it a back-handed slap and, striking one of the nævi against the calf's tooth, contused or wounded it so as to cause it to bleed a drop or two. About a week or ten days thereafter it inflamed, causing the hand and arm to swell, with rigors and considerable febrile excitement. The pustule was well formed, umbilicated and desquamated about the twenty-sixth day. All the ordinary phenomena of a well-typed bovine vaccination, and the characteristic eschar supervening, caused me to question, What is the true bovine vaccine? If the distinguished Jenner was mistaken as to how this bovine vaccine was acquired, and instead of the grease, a disease of horses' heels being the medium through the cow, it is the fresh saliva of a calf, we should experiment sufficiently to ascertain the truth."—*Maryland Medical Journal.*

**Artificial Human Milk.**—A writer in the Druggists' Circular says: I saw an article on artificial human milk, as recommended by Prof. Frankland, in the April number of your esteemed paper. I shall give you my experience on the same subject, which, I think, may save many an anxious hour to mothers unable to nurse their children. I would state that I have used this receipt for some time with the most marked success in each and every instance, the children all thriving wonderfully upon it. The last time I used it was in my own family. It is especially well adapted to regulate summer complaints of infants. My *modus operandi* is as follows: To a pint of milk add fifteen grains of saccharated pepsine, acidulated with thirty minims of dilute muriatic acid. Set the bowl containing the milk over a spirit lamp or gas burner, until it curdles solid. Then beat up the curds as fine as possible and strain through an old linen, which I found to answer the best. While straining continue to break up the curds and use some pressure to expel all the whey. To the whey received add about one-third or one-half of barley water (Decoctum Horde, U. S. P.) and sweeten the mixture to resemble human milk. It is very palatable and very nourishing. The milk should be as fresh as possible: if any cream has risen to the surface, stir the milk well.

**Mullein in Phthisis.**—Dr. F. J. B. Quinlan, (British Medical Journal) says concerning the use of this plant in phthisis, that mullein plant boiled in milk is liked by the patients; the watery infusion is disagreeable, and the succus still more so. The hot milk decoction causes a comfortable sensation, and when once patients take it they experience a physiological want, and when the supply was once or twice interrupted complained much in consequence. It eases phthisical cough; in fact, some of the patients scarcely took their cough mixture at all, an unmixed boon to phthisical sufferers with delicate stomachs. Its power of checking phthisical looseness of the bowels was very marked, and that this was not merely due to the well-known astringent properties o f boiled milk. It also gave relief to the dyspnœa. For phthisical night-sweats it was utterly useless. In advanced cases mullein does not prevent loss of weight. In pre-tubercular and early cases of pulmonary consumption, mullein appears to have a distinct weight-increasing power. In early cases mullein acts very much in the same manner as cod-liver oil; and when it is considered that it is at once cheap and palatable, it is certainly worth a trial. —*Gaillard's Med. Jour.*

**Carbolic Acid in Piles and Fistulæ.**—The fact that there is a growing tendency among physicians to the belief that carbolic acid injected into the pile tumors will radically cure and entirely remove them, and that it amounts almost to a specific in fistula, has induced Dr. A. B. Allen, of Jerseyville, Illinois, to write to the Peoria Medical Monthly for May, 1883, condemning this practice. In conclusion, he says :

"I have met over one hundred cases that had been treated by some one of the above methods before coming to me. The universal verdict has been that it is very painful, and I am certain ineffectual, and a large proportion of them certainly very much debilitated, either from the therapeutical effects of the drug, or from the excruciating pain they had undergone.—*Med. and Surg. Rep.*

**A Hydrophobia Cure.**—Dr. August Hoff writes to the Australian Medical Gazette for April: Allow me to draw your attention to a case of hydrophobia successfully treated by Dr. Offenberg, of Munster, Westphalia. The case made a great sensation, and went through all the medical and even political papers of Germany. A female peasant, twenty four years of age, was bitten by a rabid dog, and although the wouud was cauterized, the dreadful disease developed after eleven weeks. Dr. Offenberg used very energetically curare (the poison used by the Indians in the northern part of South America for arming the points of their arrows), to the extent of twenty centigrams (three and one-third grains) within five hours, although the dose for injection is usually only one-tenth to one-half grain. The patient fully recovered.—*Ex.*

**Unpleasant Effects of Quinine—Bromide of Potassium.** —Dr. J. A. Kite reports a case in which drugs could not be retained by the stomach, and a rectal injection of ten grains of bisulphate of quinine was ordered to be taken every three hours. Following the third injection, a severe headache with buzzing in the ears, accompanied by slight hallucinations. He gave an enema of thirty grains of bromide of potassium, and before fifteen minutes had elapsed all the distressing symptoms disappeared, and the patient passed into a quiet sleep.—*Med. News.*

**Hydrastin in Laryngeal Phthisis.**—Dr. Bird (Australian Medical Journal) claims good results from the treatment of laryngeal phthisis with a spray composed of hydrastin, glycerine, borax and morphia. A combination of this kind would seem likely to be of advantage.—*Buffalo Med. and Surg. Jour.*

**Removal of Warts by Cauterization.**—Dr. Cellier recommends the following treatment for warts, in the Jour. de Med. et de Chrurg. Pratiques, March, 1883: The base of the wart is transfixed by an ordinary pin, care being taken not to pierce the healthy tissue beneath. Then, the skin being protected, the head of the pin is held in the flame of a candle. In a few minutes the wart becomes white and fissured, and comes away on the point of the pin. The procedure is said to be painless as well as bloodless. The curious assertion is made by Dr. Cellier that it is necessary to remove but one wart on the hand, and all the others (sometimes even a dozen or more) will disappear without treatment—*Med. Record.*

**The Treatment of Croup.**—Dr. Charles J. Fahie writes to the British Medical Journal, May 12, 1883, that out of ten cases of croup treated as follows, he did not lose one. He provides that the case must be seen early. A hot bath, a hot poultice of salt to the throat externally, a mustard emetic, and a dose (to be regulated according to the age of the child) of the following mixture every two hours: Tartar emetic, liquor ammoniæ acetatis, and mistura citratis potassæ, to six ounces. The citrate of potash mixture can be made by saturating bicarbonate of potash with citric acid.

Such success raises a question as to the correctness of Dr. Fahie's diagnosis, about which he says nothing.—*Med. and Surg. Rep.*

**Swallowing of Shot and Insufflation in the Treatment of Ileus.**—From the London Medical Record, May 15, 1883, we learn that in three cases (Gazz. Med. Ital. Lomb, February 10, 1883), with well-marked symptoms of invagination of the bowel, obstinate constipation, stercoraceous vomiting, pain, etc., Dr. Pedrini, after other remedies had failed to relieve, made the patient swallow five or six bullets and two kilogrammes of No. 3 shot, at the same time using prolonged and repeated insufflation of air by the rectum. In each case the success of this treatment was complete, relief being quickly obtained, and the patient making a good recovery.—*Med. and Surg. Rep.*

**Glycerine.**—M. Desguin, of Anvers, has given glycerine internally in certain forms of skin disease with, it is said, marked success, especially in acne punctata and the furuncular diathesis. He commences with four drachms daily and gradually increases the dose. He states that the secretion of the cutaneous glands, which is thick and irritating in these diseases, becomes more liquid, and cutaneous irritation is notably lessened. During the convalescence from scarlet fever he believes that it facilitates desquamation.—*Lancet. Southern Practitioner.*

**Lancing the Gums.**—Dr. Chase, in the Dental Journal, gives the following practical advice bearing on this trifling but often quite important operation:

The operator should know whether a tooth is pressing on the gum, and trying to make its way out. In this case, cut down to the new tooth, until it is felt under the lancet. For incisors and cuspids, a straight line cut, for molars, a cross-cut.

How not to do it: With a child sitting up, in your lap, or any one's lap.

How to do it: Let the operator and "nurse" sit close together, facing each other. The child is laid down, face upward; the head in the operator's lap, the feet in the "nurse's" lap. The nurse holds the limbs of the child quietly, so that it may not interfere.

With the left hand the operator takes the jaw between his fingers, and slowly and firmly does the cutting. There is no false cut. The child is still.—*Med. Age.*

**How Long Should the Subjects of Contagious Diseases be Isolated?**—The Academy of Medicine of Paris, after a careful study and report of a special commission, has given the following answer to the above inquiry.—(*Gaz. Med. de Paris.*)

1. Pupils affected with chicken-pox, small-pox, scarlet fever, measles, mumps or diphtheria, should be strictly isolated from their comrades.

2. For small-pox, scarlet fever, measles and diphtheria, isolation should not be shorter than forty days; for chicken-pox and mumps twenty-five days is enough.

3. Isolation should last until after the patient has been bathed.

4. The clothing worn by the patient at the time he was taken sick should be subjected to a temperature of 90° C. (194° Fahr.), and to sulphur vapor, and then well scoured.

5. The bedding, curtains and furniture should be thoroughly disinfected, washed and aired.

6. The pupil of a school, after recovery from one of the above contagious diseases should not be readmitted to the school unless furnished with the certificate of a physician that the above precautions have been observed.—*Can. Lan.*, *Mar.*

**Death from Petroleum by Suffocation.**—During the night from September 18 to 19, 1882, twelve young girls died in the institution Cavaller Maggiore in Piedmont the death of suffocation, because they had permitted a kerosene lamp to burn during the night after they had turned it half down. The flame evidently communicated itself to the fluid in the lamp, and gradually abstracted all oxygen from the room, which fact was the cause of the suffocation. The dead bodies showed all the signs of death by suffocation.—*Med. and Surg. Rep.*

**Quinine Intoxication.**—A writer in the last number Die Pharmaceutische Post, says that as a remedy for the relief of quinine intoxication, as he calls the over stimulation caused by quinine in excessive doses, he has used ergot in several cases and finds that to neutralize the cerebral effect of one gramme of quinine at least one and a half grammes of powdered ergot or one gramme of ergotin must be employed. With this remedy the most annoying tinnitus may be entirely removed during the administration of quinine.—*Quinologist.*

**Baer's Tonic.**—The following is Dr. Baer's favorite tonic for diseases of women :

| | | |
|---|---|---|
| ℞ | Corrosive sublimate | gr. j, |
| | Tr. iron muriate, | ʒ iv, |
| | Acid hydrochloric | ʒ iv, |
| | Solution chloride arsenic | ʒ j, |
| | Syrup | fl. ℥ ijss, |
| | Water, q. s. | fl. ℥ vj. |

M. Sig. Two teaspoonfuls three times a day after meals.

—*Medical Herald.*

**Turpentine in Diphtheria.**--A German apothecary, R. Numch, of good reputation among those who know him, reports that in the case of his own seven-year-old daughter a teaspoonful night and morning of oleum terebinthinæ purificatum effected a cure of diphtheria. Others who have observed the action of the drug report that its effects are wonderful, a bright redness beginning to spread from the margin of the exudation within half an hour after its administration and, becoming generally diffused, takes the place of the false membrane in twenty-four hours. Although its effects are most marked early in the disease, it is said to be also valuable, although acting less quickly, after several days have elapsed. It may be mixed with tepid milk. The dose of an adult is a tablespoonful. The remedy is certainly a simple one, and, tried early in the disease, its employment need not prevent other treatment if it fail.—*Medical Age, March 10.*

**Sub-mucous Chloroform Injections in Toothache.**—Gaillard's Medical Journal, May 19, 1883, says that Dr. Guillot (Progress Medical, March 24, 1883), claims to have had very good results in the treatment of toothache from the injection of chloroform beneath the mucous membrane of the gums. The effects are more immediate and lasting than those of morphine. There have been no resultant abscesses or inflammations.—*Med. and Surg. Rep.*

**Medical Treatment of Obstinate Neuralgia.**—M. Verneuil, in a communication to the Surgical Society of Paris (Le Prog. Med., No. 49, 1882), referring to the surgical treatment of obstinate neuralgia, said that all therapeutic resources should be exhausted before surgical interference was undertaken. He recalled a case which was cured by hyoscyamin, after resection of all the ends of nerves and even amputation had failed to give relief.—*Medical Record.*

**A Smart Doctor.**—An old wealthy sinner had yawned his jaw out of joint and came to a doctor for help. The doctor "set" the jaw and the patient refused to pay but half the bill. The doctor told a funny story, at which the patient laughed very heartily, and out went the old man's jaw again. This time the doctor secured his fees for both operations before performing his work, but the patient "never smiled again."—*Home Health.*

